# Abiotic and Biotic Damage of Microalgae Generate Different Volatile Organic Compounds (VOCs) for Early Diagnosis of Algal Cultures for Biofuel Production

**DOI:** 10.3390/metabo11100707

**Published:** 2021-10-15

**Authors:** Kristen L. Reese, Carolyn L. Fisher, Pamela D. Lane, James D. Jaryenneh, A. Daniel Jones, Matthias Frank, Todd W. Lane

**Affiliations:** 1 Biosciences and Biotechnology Division, Physical & Life Sciences Directorate, Lawrence Livermore National Laboratory, Livermore, CA 94550, USA; kleighreese@gmail.com (K.L.R.); frank1@llnl.gov (M.F.); 2 Forensic Science Center, Nuclear and Chemical Sciences Division, Lawrence Livermore National Laboratory, Livermore, CA 94550, USA; fisher77@llnl.gov; 3 Systems Biology Department, Sandia National Laboratories, P.O. Box 969, Livermore, CA 94550, USA; plane@sandia.gov (P.D.L.); jdjarye@sandia.gov (J.D.J.); 4 Department of Biochemistry and Molecular Biology, Michigan State University, East Lansing, MI 48824, USA; jonesar4@msu.edu; 5 Bioresource and Environmental Security Department, Sandia National Laboratories, P.O. Box 969, Livermore, CA 94550, USA

**Keywords:** *Microchloropsis gaditana*, *Brachionus plicatilis*, volatile organic compounds, SPME-GCMS

## Abstract

Open microalgal ponds used in industrial biomass production are susceptible to a number of biotic and abiotic environmental stressors (e.g., grazers, pathogens, pH, temperature, etc.) resulting in pond crashes with high economic costs. Identification of signature chemicals to aid in rapid, non-invasive, and accurate identification of the stressors would facilitate targeted and effective treatment to save the algal crop from a catastrophic crash. Specifically, we were interested in identifying volatile organic compounds (VOCs) that can be used to as an early diagnostic for algal crop damage. Cultures of *Microchloropsis gaditana* were subjected to two forms of algal crop damage: (1) active grazing by the marine rotifer, *Brachionus plicatilis*, or (2) repeated freeze–thaw cycles. VOCs emitted above the headspace of these algal cultures were collected using fieldable solid phase microextraction (SPME) fibers. An untargeted analysis and identification of VOCs was conducted using gas chromatography-mass spectrometry (GC-MS). Diagnostic VOCs unique to each algal crop damage mechanism were identified. Active rotifer grazing of *M. gaditana* was characterized by the appearance of carotenoid degradation products, including β-cyclocitral and various alkenes. Freeze–thaw algae produced a different set of VOCs, including palmitoleic acid. Both rotifer grazing and freeze–thawed algae produced β-ionone as a VOC, possibly suggesting a common stress-induced cellular mechanism. Importantly, these identified VOCs were all absent from healthy algal cultures of *M. gaditana*. Early detection of biotic or abiotic environmental stressors will facilitate early diagnosis and application of targeted treatments to prevent algal pond crashes. Thus, our work further supports the use of VOCs for monitoring the health of algal ponds to ultimately enhance algal crop yields for production of biofuel.

## 1. Introduction

Over the next two decades, the world’s human population is projected to increase by 1.3 billion [1]. Recently industrialized, developing nations with improved standards of living will drive higher energy consumption. Based on these projections, the International Energy Agency (IEA) forecasts a 19% increase in world energy demand by the year 2040 [1]. To meet these future energy demands, microalgae are a promising prospect for biofuel production [2]. Eukaryotic microalgae are a diverse group of organisms that can grow in a variety of environmental conditions, such as sewage, wastewater, alkaline, brackish, and saline water systems [3]. Production of microalgae does not require arable land and thrives outdoors in desert areas with natural sunlight. Thus, it is believed that microalgae may represent a viable path to create renewable transportation fuels and feedstocks to better support growing global energy needs.

Although many species and strains of microalgae have been tested for use in biofuel production, relatively few have been found to be both high lipid producers and amenable to large-scale industrial production with natural sunlight [4]. One promising genus for biofuel production is *Microchloropsis* (formerly *Nannochloropsis*) [5], whose members have been found to have up to have ~40% of their biomass as lipids and exhibit high productivity in outdoor mass production systems with natural sunlight [6]. Additionally, *Microchloropsis gaditana* is genetically tractable, meaning genetic engineering and optimization are more likely to be successful [3,7], and has been extensively characterized in terms of flocculation, extraction of bioenergetic molecules [8]. Thus, *M. gaditana* is an important candidate for a biofuel production species [9,10].

Algal production systems are subject to both abiotic and biotic threats resulting in pond crashes with high economic costs. Abiotic threats include depletion of a nutrient, oxygen deficiency, overheating, freezing, pH disturbance, or contamination [11]. Many algal species are sensitive to freezing which results in a physical disruption of the cell through the formation of ice crystals [12]. Biotic threats for biofuel production from microalgae is the wide variety of pathogens, grazers, parasites, and competitor species that are detrimental to algal growth and production [13]. As part of their study to define reliability metrics for algal cultivation systems, Harmon et al. [14] have defined pond failure or crash as “when pond operational systems, human error, biological contamination, and/or abiotic stresses cause the algae in the pond to manifest reduced growth rates or completely cease growing as observed by stagnation or a decrease in optical density (OD), or in extreme cases, death of the algae culture resulting in a complete clearing of the pond”. Conservatively, it is estimated that 30% of algal pond crashes are the result of biological contaminants [15]. Developing methodology to monitor, circumvent, and ultimately prevent predation of algae will increase biomass production, drive down costs for algae farmers, and reduce the risk involved with algae cultivation, making it more favorable for investment by entrepreneurs and biotechnology companies. Current methods to monitor and mitigate unhealthy ponds exist, but intervention before culture collapse occurs is hindered by laborious procedures, data with low signal-to-noise, and a lack of early indicators. For example, light microscopy is a standard method for monitoring pond samples for biological contaminants [16]; however, it is limited by the advanced training needed to differentiate various microbial species, the time-consuming and labor-intensive work of analyzing samples “by eye”, and a challenge with acquiring a representative sample of the entire culture (e.g., analysis of a 10 mL pond sample from a 100,000 L algal production pond likely will not capture all types of pond inhabitants). For these reasons, imaging flow cytometry [17,18], and nucleic-acid hybridization techniques [19] are under study to automate, simplify, and expediate the pond contaminant monitoring process.

As an alternative, non-invasive, and diagnostic pond-monitoring method, we and others are interested in identifying signature chemicals to aid in rapid and accurate identification and/or detection of biological contamination in order to facilitate targeted and effective treatment to save the algal crop from a catastrophic crash. It is well known that deleterious species produce various volatile organic compounds (VOCs) as specific biomarkers of their predatory action [20] and monitoring VOCs in health and disease is similarly underway [21]. Since microalgae and their predators both produce volatile organic compounds (VOCs) throughout their life cycle [22,23], there is potential that VOCs serve as indicators of unhealthy ponds that may indicate the presence of microalgal predators. Recently, we have demonstrated feasibility of using VOCs as biomarkers of pond health with *Microchloropsis salina* in the presence and absence of the marine rotifer, *Brachionus plicatilis* [24,25]. These works used solid-phase microextraction (SPME) or Carbopack thermal desorption (TD) tubes to non-invasively collect headspace volatiles for analysis by gas chromatography-mass spectrometry (GC-MS).

In the present study, we expand upon this work by determining if VOCs generated by the algae undergoing grazing by rotifers are specific for the grazing event or represent a generic chemical signal of algae crop damage. The goal of this study was to determine the difference between the VOCs released from algae upon ingestion by a rotifer, with all the attendant digestive processes, and those released by the physical disruption of the algae alone. Grazing by rotifers was selected as the biotic crash mechanism because they are important threat to production systems with which we have significant experience and have developed standard grazer assays. Among the various abiotic crash mechanisms, freeze thaw treatment was selected because of the ability to treat the entire algal culture to ensure that the vast majority of cells were disrupted simultaneously. Second, it was not necessary to alter the physiological state for the algal cells (i.e., through nutrient depletion) to achieve physical disruption. That allowed us to match the physiological state of the culture prior to freeze–thaw to that of the culture prior to rotifers addition.

In this study we have surveyed and compared the production of VOCs by *M. gaditana* in the presence and absence of both abiotic and biotic damage. We have identified specific VOC differences for *M. gaditana* in the presence of rotifer grazing versus heathy cultures. Although several VOCs are the same as produced by *M. salina* in the presence of actively grazing rotifers [24], we have identified at least one reportable difference. Additionally, we induced the physical disruption of *M. gaditana* cells through repeated freeze–thaw events which elicited a different array of VOCs, suggesting that different algal crop damaging events might produce specific VOCs that could be used diagnostically and aid in specific interdictive strategies to save a pond from crashing [4]. Resulting chemical fingerprints are potential targets for future chemical monitoring systems (such as micro-GC device, see [26] for review) in microalgal cultivation.

## 2. Results

Our experimental setup facilitated monitoring of headspace VOCs from *M. gaditana* over the course of two different types of algal crop damage mechanisms: (1) grazing of *M. gaditana* by the marine rotifer, *B. plicatilis*, and (2) freeze–thawing of *M. gaditana*. In the rotifer grazing versus freeze thaw experiments we were measuring two different processes, reduction in cell number due to grazing and physical disruption, but not reduction of cell number, due to freeze–thaw. Separate methods were used to were used to monitor each of these processes. Direct enumeration (i.e., cell counting) is the least ambiguous way of measuring biomass loss through grazing as fecal pellets such as those produced by rotifers do retain some chlorophyll fluorescence but not the physical dimensions of intact algal cells. However, this direct determination of biomass concentration proved to be incompatible with freeze–thaw treatments that resulted in the rupture of the cells and loss of cellular constituents without resulting in a decrease in particle number as the cells retained their overall physical structure to a sufficient degree for counting. Fluorescence measurements demonstrated that the culture was clearly nonviable (did not increase in concentration) but rather lost color presumably due to the degradation of the released chlorophyll.

### 2.1. Monitoring Algal Concentration during Rotifer-Grazing and Freeze–Thaw Experiments

#### 2.1.1. Rotifer-Grazing Experiments

**Figure 1:**
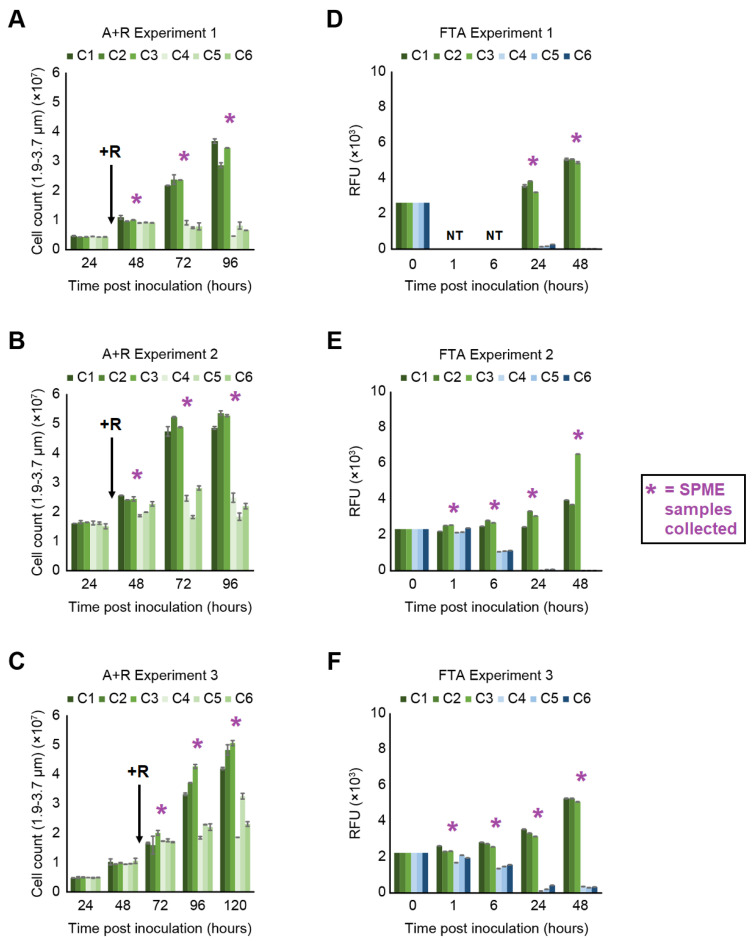
(**A**–**C**) Algae concentrations as determined by direct enumeration for three healthy algae cultures (C1–C3) and three rotifer grazing algae cultures (C4–C6) for the three A + R experiments. Rotifer addition marked by “+R” with an arrow at the relevant point in time. (**D**–**F**) Fluorescence measurements for three healthy algae cultures (C1–C3) and three freeze–thawed algae cultures (C4–C6) for the three Freeze–Thaw Algae (FTA) experiments. The error bars represent standard deviation calculated from technical replicates for each sample (*n* = 2). The starting concentrations of the FTA experiments were equivalent at the time of algal inoculation (t = 0 h), and thus the standard deviation for these measurements is zero. No timepoints for1 h and 6 h in FTA Experiment 1 were taken, as denoted by “NT”. SPME samples were collected at timepoints in experiments marked by an asterisk (*).

Algal cell concentrations for cultures of *M. gaditana* in the presence or absence of grazing *B. plicatilis* were measured at several timepoints for *M. gaditana* alone (abbreviated Algae or A), *M. gaditana* and *B. plicatilis* (abbreviated Algae + Rotifer or A + R), and ESAW media blanks (abbreviated media blanks or MB), as a negative control (Figure 1A–C). After *B. plicatilis* was added to three *M. gaditana* cultures (Figure 1A–C, Cultures C4–C6), time-dependent decreases in algal density were observed relative to the *M. gaditana* only controls (Figure 1A–C, Cultures C1–C3). For all three A + R experiments, rotifer grazing slowly depleted algal concentration over a period of several days (Figure 1A–C, Cultures C4–C6). Resulting A + R cultures had roughly 20–50% fewer intact algal cells present compared to corresponding time-matched A cultures. A + R cultures between the three experiments displayed different rates of algal biomass loss, where we attribute variation in rates of biomass loss to differences in the commercially supplied rotifer lots.

#### 2.1.2. Freeze–Thaw Experiments

Algal cell concentrations were likewise monitored daily, however chlorophyll-based fluorescence measurements, instead of direct enumeration via cell counting, was used for cultures of *M. gaditana* exposed to three successive freeze–thaw cycles (abbreviated Freeze–Thaw Algae or FTA) (Figure 1D–F) and ESAW media blanks as a negative control. This alternative algal-density determination method was employed because with FTA Experiment 1, it was observed that direct enumeration was not a reliable way to determine the quantity of viable microalgal cells still present in the culture (data not shown). Thus, for those cultures undergoing the freeze–thaw process (Figure 1D–F, Cultures 4–6), time-dependent decreases in chlorophyll fluorescence signal relative to actively growing *M. gaditana* cultures (Figure 1D–F, Cultures 1–3) were observed. Resulting FTA cultures displayed a rapid decrease in fluorescence signal compared to corresponding time-matched healthy algae cultures, on average displaying ~60% of the relative fluorescence signal after 6 h (Figure 1E,F) and less than 10% of the relative fluorescence signal after 24 h of growth and beyond (Figure 1D–F). For this reason, the experiments were ended after 48 h, instead of 96 or 120 h as for the A + R Experiments.

### 2.2. Monitoring VOC Emissions during Rotifer-Grazing and Freeze–Thaw Experiments

For all experiments, data outputs from SPME-GC-MS were subjected to similar chromatographic deconvolution user-defined parameters established previously for this type of analysis [24]. The number of volatile compounds detected within each sample varied from ~100–200. Potential biomarkers for rotifer grazing were selected as being ‘unique’ to a wounding condition and distinct from the healthy controls through the application of filtering criteria, which were based upon a VOC’s relative abundance and detection frequency across experimental replicates. Within a single experiment (e.g., A + R Experiment 1), the filtering criteria narrowed the list of thousands of VOCs detected down to less than 40. From here, the individual experiments (e.g., A + R Experiment 1, 2, and 3) were compared and putative biomarkers were identified, as previously defined, as being present in at least 2 of the 3 replicate experiments (summarized in Table 1 and Table 2). VOCs not meeting the full criteria as a putative biomarker are summarized in Supplemental Tables S1 and S2.

#### 2.2.1. Rotifer-Grazing Experiments

Grazing of *M. gaditana* by *B. plicatilis* produced six putative biomarkers, enumerated R1-R6 (Table 1). Three biomarkers, Compounds R1, R3, and R4, were identified with an 83%, 75%, and 80% confidence scores to the chemicals β-cyclocitral, 4-(2,6,6-trimethyl-1-cyclohexen-1-yl)-2-butanone, and β-ionone, respectively. Compounds R5 and R6 were tentatively identified with a 71% and 78% confidence to the alkenes 1-pentadecene and 1-heptadecene, respectively.

Reference standards of β-ionone and β-cyclocitral were analyzed using the GC-MS method described and confirmed as the identities of Compound R4 (Table 1) and R1 (Table 1), respectively, using both retention index matching and comparison of MS fragmentation patterns. The retention index for the analytical standard of β-ionone (1495) was <1% different from that of the experimentally observed biomarker R4 (1494) and the NIST literature RI (1486). The retention index for the analytical standard of β-cyclocitral (1215) was also <1% different than that of the experimentally observed biomarker (1208) and the NIST literature RI (1220).

#### 2.2.2. Freeze–Thaw Experiments

A total of over 3200 VOCs were detected within the three FTA replicate experiments and application of filtering criteria narrowed this down to less than 100 that were reproducibly present across multiple experiments. Of those VOCs reproducibly detected, 10 putative biomarkers were identified for the FTA experiments and are labeled as Compounds F1-F10 in Table 2. The Compounds F7 and F9 were tentatively identified with a 93% and 80% confidence to β-ionone and palmitoleic acid, respectively.

#### 2.2.3. Comparison of Algae Crop Damage Mechanisms

**Figure 2:**
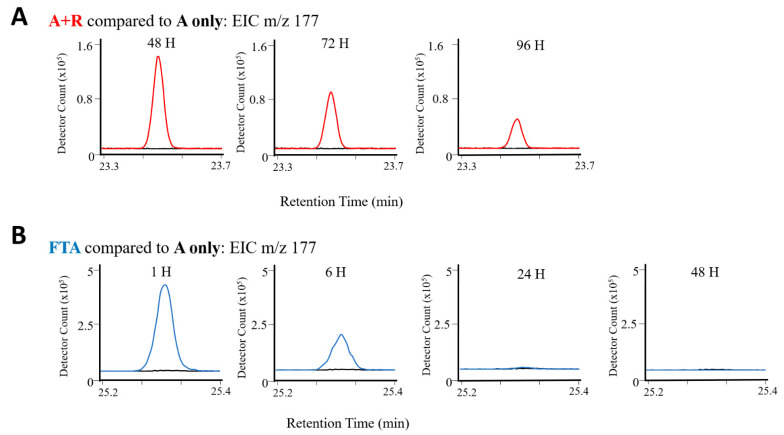
Example extracted ion chromatograms for *m/z* 177 for putative biomarker, β-ionone, observed in both rotifer-grazed *M. gaditana* (A + R, red) and time-matched healthy algae controls (A only, black) in part (**A**) and freeze–thawed *M. gaditana* (FTA, blue) with time-matched healthy algae controls (A only, black) in part (**B**) throughout the duration of condition-specific timepoints measured during each experiment.

**Figure 3:**
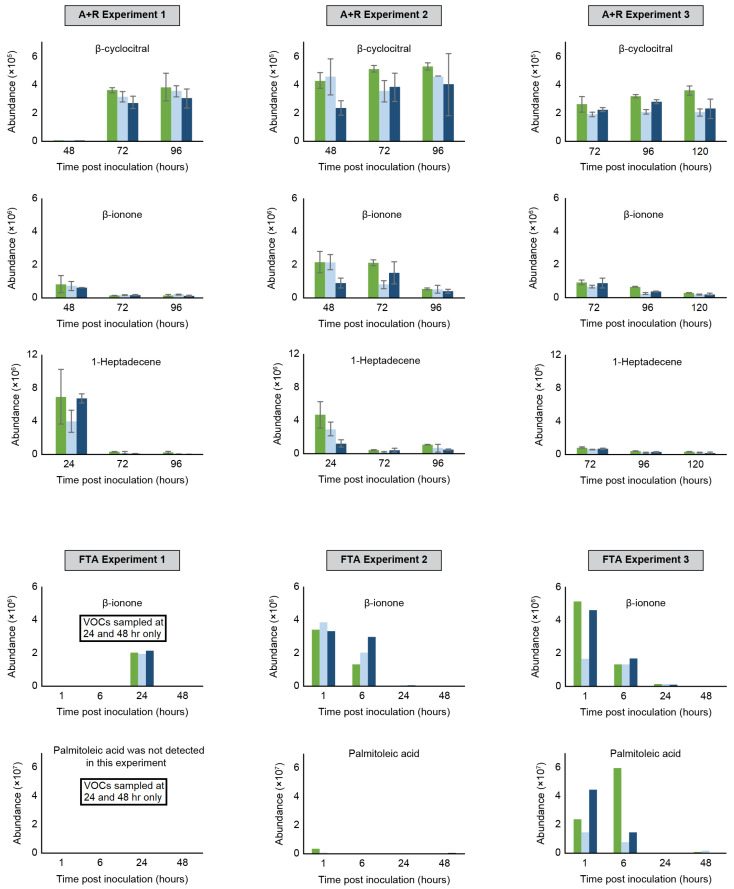
Peak areas of extracted ion chromatograms for β-cyclocitral, β-ionone, and 1-heptadecane in each A + R replicate experiment (top nine graphs) and for β-ionone and palmitoleic acid in each FTA replicate experiment (bottom six graphs). Error bars in A + R experiments represent standard deviation of technical replicates for each sample. Semi-quantitative VOC analysis for the same putative biomarker was set relative to the highest intensity across all three replicate experiments. For comparison across A + R and FTA experiments, relative abundance scale for β-ionone was set relative to the observed highest peak area in FTA Experiment 3.

β-ionone was monitored in both A + R and FTA experiments using extracted ion chromatograms for its base peak, *m/z* 177 in Figure 2. The rotifer-grazing VOC profiles (Figure 2A) display maximum β-ionone signals of ~7 × 10^5^ at the first timepoint measured after rotifer addition (48 h post-inoculation). The signal decreased to an average of 58% and 27% of the original signal after 24 and 48 h, respectively, with a final peak area averaging ~1 × 10^5^. A time-matched monitoring of healthy algae cultures (Figure 2A) from the rotifer grazing experiments showed no detectable signal of *m/z* 177, and presumably β-ionone for all timepoints sampled. For FTA, represented in Figure 2B, an average signal of ~3 × 10^6^ was observed at the first timepoint ~1 h post-inoculation. The signal rapidly decreased over the subsequent timepoints, averaging 54% and 3% of the original signal at 6- and 24-h post-inoculation, before becoming undetectable at the final timepoint of 48 h post-inoculation. As with the A + R experiments, time-matched health algae controls (A only) for FTA experiments also showed no detectable signal of *m/z* 177, and presumably β-ionone, for all timepoints monitored. Differences in algal crop damage methods were evidenced by unique VOC profiles, summarized in Figure 3. In the A + R cultures, the presence of β-cyclocitral, β-ionone, and 1-heptadecene were detected at three timepoints only in samples that had rotifers present. The relative abundance of these compounds either remained consistent (β-cyclocitral) or gradually decreased with continued rotifer grazing (β-ionone and 1-heptadecene). All three putative biomarkers were still detected at the final A + R timepoint. Conversely, β-ionone and palmitoleic acid were only detected within the first 6 h after the freeze–thawed algal cells were added to the media.

## 3. Discussion

The headspace VOCs were monitored from *M. gaditana* being actively grazed by the marine rotifer, *B. plicatilis*. During grazing, a single rotifer can consume ~200 algal cells per min [27] and the ultimate depletion of the algal culture is dependent on the initial concentration of the algal inoculum and the number of rotifers added. In addition to algae undergoing active grazing, viable and actively growing algae were also present in these cultures. Thus, algal growth rates and VOC emissions were detected during this time for both healthy and grazed algae. The initial algal concentrations were chosen to allow the grazing to take place over several days for the collection of multiple time-resolved VOC samples.

Algal cell concentrations were monitored for cultures of *M. gaditana* exposed to three successive freeze–thaw cycles. During this process, algal cells are physically disrupted and rendered non-viable through the formation of ice crystals. This is evidenced by the lack of growth and release of photosynthetic pigments from the cells. All algae cells were simultaneously frozen and lysed within a comparatively shorter time frame compared to rotifer grazing, preventing active growth after freeze–thaw procedure was complete. The direct enumeration of the FTA cultures, by particle counting, showed no decrease in particle number but FTA cultures displayed a rapid loss of chlorophyll color and fluorescence due to a release and apparent degradation of chlorophyll from disrupted cells when compared to time-matched healthy A controls. Additionally, since the FTA cultures never increased in algal concentration over the course of the FTA experiments, it can be inferred that the entire algal culture was, upon treatment, disrupted and rendered nonviable. Thus, we utilized chlorophyll fluorescence to demonstrate the lack of algal culture growth [28].

The cellular disruption of *M. gaditana* cells was accomplished via repetitive freeze–thaw cycles to abiotically result in algal cell death via rapid ice crystal formation. The freezing process quickly affects the entire algal culture, simultaneously, compared to gradual algal crop damage from rotifer grazing. During FTA Experiment 1, measurements of FTA VOCs at 24 and 48 h indicated that VOCs generated by the freeze–thaw cycle were mostly depleted by 24 h and not detected at 48 h post-inoculation. After the FTA Experiment 1 (Figure 1D), the experimental design was altered to collect VOCs at additional timepoints (e.g., 1 h and 6 h) post algal inoculation (Figure 1E,F). In doing so, we observed a higher diversity of VOCs generated by the freeze–thawed algal samples in subsequent Experiments 2 and 3.

The fundamental difference between the two crop damage mechanisms is that freeze thaw is strictly a physical process whereas grazing has a biological component in the rotifer digestive tract. The digestive processes of the *B. plicatilis* are not well characterized. It is known that at higher prey concentrations, as would be expected in high density algae production ponds, ingested particles travel faster thought the rotifer digestive track and may not be completely digested. Although relatively little is known of the rotifer digestive process along with those of other microzooplanktonic grazers it is not unexpected that the VOCs released from grazing would differ from those released from purely physical disruption. However, our data reveals that some VOCs are released by both processes.

β-ionone, generated from the breakdown of carotenoids, was the only biomarker identified as a product of both freeze–thaw disruption and rotifer-grazing (Compound R4). β-ionone was seen previously in rotifer-grazed cultures of *M. salina* [24]. β-ionone gradually decreased (<96 h) with rotifer grazing and rapidly decreased (<6 h) after initial freeze–thawing. The prolonged detection of β-ionone in the rotifer-grazing is consistent with algal cells being gradually individually consumed by rotifers with concomitant oxidative degradation of carotene. This slower process results in the continuous release of VOCs over a period of days. Conversely, a sudden, initial burst of β-ionone, followed by rapid deterioration of signal afterwards, is consistent with the rapid release of cellular contents from repeatedly freeze–thawed algal cells, which occurs over the span of minutes or hours. The detection of β-ionone indicates that the formation of this carotenoid breakdown product may not be dependent on the rotifer digestion process but may be a product of abiotic processes that are held in common.

Palmitoleic acid was detected in the headspace of freeze–thaw cultures only and was likely released as a result of the physical disruption of the algal. Palmitoleic acid, is a monounsaturated fatty acid (MUFA) and an ideal candidate for biofuel production [29]. As a desirable product of algal cultivation, it has been shown that algae generate higher cellular levels of palmitoleic acid under abiotic stress conditions, such as nutrient limitation [30,31]. *M. gaditana* is routinely studied as a food source for the aquaculture production of rotifers such as *B. plicatilis* and it is known that the fatty acid content of the feed algae has a significant impact on the biochemical composition of the rotifers [32]. It seems likely that in the case of rotifer grazing the palmitoleic acid present in the microalgae is utilized by the rotifer rather than escaping to the volatile fraction. Palmitoleic acid derived from algal extract has been used to mitigate inflammation induced within a human macrophage cell line [33].

Several VOCs were detected specifically during the process of rotifer grazing. Along with β-ionone, grazed cultures of *M. gaditana* released the biomarkers β-cyclocitral and 4-(2,6,6-trimethyl-1-cyclohexen-1-yl)-2-butanone. These structurally related ketones or aldehydes are known derivatives of carotenoid oxidation [34,35,36,37]. We have previously observed carotenoid degradation products as indicators of rotifer grazing of the closely related algal species, *Microchloropsis salina* [24]. We hypothesize that these compounds, that were not detected under conditions of abiotic physical wounding, may represent further degradation of the part of the carotenoid pool. *M. salina* and *M. gaditana* are phylogenetically related [5], thus observance of the same putative biomarkers is not surprising. Previously, other microalgal strains have been shown to emit the some of the same VOCs during algal wounding [38]. In addition to the obvious carotenoid breakdown products both 1-pentadecene and 1-heptadecene were detected during rotifer grazing only. The presence of the long-chain alkenes could be due to the oxidation of β-carotene or long-chain fatty acids—both of which are highly abundant in oleaginous algal strains, such as *M. gaditana* [39].

Our results clearly show differences in the VOCs produced by physical disruption and those produced by grazing and contact with the rotifer digestive system. This work indicates that it may be possible to develop biomarkers that are indicative of different algal stress or damage.

## 4. Materials and Methods

### 4.1. Microalgae and Rotifer Cultures

Axenic *Microchloropsis gaditana* CCMP 526 was obtained from National Center for Marine Algae and Microbiota (NCMA at Bigelow Laboratory, East Boothbay, ME, USA). Axenicity of the culture was defined by NCMA standards and *M. gaditana* cultures were not further tested in house. *M. gaditana* cultures were grown in modified ESAW medium [40] made with 7.5 mM NaNO_3_ and 0.5 mM Na_3_PO_4_ and MilliQ water. Stock cultures of *M. gaditana* were grown at 20 °C, a light intensity of 100 µmol m^−2^s^−1^ and a 16:8 h light:dark cycle. Volatilomics experiments were conducted under the same conditions as in Reese et al. 2019 [24], except 80:20 N_2_/O_2_ was used as the VOC-free research grade air with a purity of 99.999% (Matheson Tri-Gas, West Sacramento, CA, USA). Briefly, cultures were continuously sparged with 1% CO_2_ and 99% air for a total mass flow of 900 cc min^−1^ split equally across the six culture vessels (150 cc min^−1^ sparging rate for each individual sample). Xenic rotifer cultures of *Brachionus plicatilis* (Reed Mariculture, Campbell, CA, USA) were concentrated, counted, and added to experiments in the same way as described in [24].

Cultivation of *M. gaditana* and *B. plicatilis* at 15-L scale was carried out according to the methods described in [24] with few differences, detailed herein. All three experiments of *M. gaditana* and *B. plicatilis* (referred to as “A + R Experiments 1–3”, or “A + R 1–3”) utilized six 20-L carboys. Prior to algal inoculation, 15 L of ESAW media were 0.2 µm-filtered, added to each of the six carboys, and then media blank headspace samples were collected for 1 h on SPME fibers (described further below). All six carboys were then inoculated with *M. gaditana* to a final concentration of 5–6 × 10^6^ cells mL^−1^ for A + R Experiments 1 and 3, and 12 × 10^6^ *M. gaditana* cells mL^−1^ for A + R Experiment 2. For all experiments, microalgae were grown under the same conditions as described in Reese et al. [24]. (Briefly: 2000 µmol m^−2^s^−1^ of 24 h light, 22–25 °C, 150 cc min^−1^ sparging of 1% CO_2_ through bubbler). For all A + R Experiments, 6.6 × 10^5^ live rotifers (final concentration of 44 rotifers mL^−1^) were added to three of the six *M. gaditana* cultures (carboys 4, 5, and 6, abbreviated C4, C5, C6). For A + R Experiments 1 and 2, the rotifers were added to the algae cultures 24 h after algae inoculation and for A + R Experiment 3, the rotifers were added to the algae 48 h after algae inoculation. Rotifers were concentrated, counted, and prepared for addition to cultures as described previously [24]. Fewer rotifers were used for these experiments, in comparison to Reese et al. [24], due to the slightly smaller size of the *M. gaditana* cells and presumed higher grazing rate by *B. plicatilis*.

### 4.2. Freeze–Thaw Algal Experiments

Freeze–Thawed Algae (FTA) experienced cellular disruption in the absence of rotifers by repeatedly freeze–thawing concentrated *M. gaditana* before adding to media (these experiments are referred to as “FTA Experiments 1–3”, or “FTA 1–3”). These experiments had a similar setup (e.g., ESAW media, CO_2_/air sparging rates, initial algal concentration) as the previously described algae + rotifer experiments except that the final culture volume for each of the six replicates was 900 mL, held in glass 1-L Pyrex containers. After media blank headspace samples were collected by SPME (as described previously for algae + rotifer experiments), *M. gaditana* inoculum was concentrated by gentle centrifugation (2500× *g*) down to ~100–120 mL and then evenly divided between six conical tubes for each of the six Pyrex containers in the experimental setup. Three of these conical tubes (each containing ~15–20 mL of concentrated *M. gaditana*) underwent three of the following freeze–thaw cycles: frozen for 15 min in a slurry of 100% ethanol and dry ice and then thawed for 10 min in water heated to 42 °C with intermittent vortexing. After three of these freeze–thaw cycles were complete, all six aliquots of algae (three control cultures and three freeze–thawed cultures) were added to each of the six 900 mL volumes of ESAW media, resulting in all six cultures starting with final concentrations of 10 M cells mL^−1^ for FTA Experiment 1, 9 M cells mL^−1^ for FTA Experiment 2, and 8.5 M cells mL^−1^ for FTA Experiment 3.

### 4.3. Daily Timepoints to Monitor Viability of Algal Cultures for A + R and FTA Experiments

For A + R experiments, algal concentration was determined daily by direct enumeration (Coulter Counter; Beckman Coulter, Indianapolis, IN, USA) of algal cells, as done previously [24]. For FTA experiments, the freeze–thaw did not result in total destruction of the cells but rather leakage of the cellular contents into the medium. Thus, particle counting did not discriminate between live and dead cells, and particle number remained constant through the experiment (data not shown). Chlorophyll fluorescence provided a measure of chlorophyll destruction. We utilized the standard method for monitoring chlorophyll fluorescence (430 nm excitation, 685 nm emission) using Tecan i-control infinite 200 Pro, version 1.11.1.0, plate reader. Both direct enumeration (i.e., particle counting) and chlorophyll fluorescence were indicative of algal cell death.

### 4.4. SPME Headspace Sampling and GC-MS Data Acquisition

VOCs from the headspace of each culture and media control were collected using portable field sampler solid-phase microextraction (SPME) fibers at the same time as algal density measurements. The methodology of VOC sampling was the same for the algal damage occurring via rotifer grazing (A + R Experiments 1–3) and or freeze–thaw cellular disruption (FTA Experiments 1–3). The collection procedures were similar to those described in Reese et al. [24], briefly summarized here with relevant modifications. Bi-phasic coated SPME fibers with 65 µm polydimethylsiloxane/divinylbenzene (PDMS/DVB) coatings (Supelco, Bellefonte, PA, USA) were introduced to the headspace above culture vessels for 60 min during relevant experimental timepoints. Three vessels were prepared as biological replicates. For the A + R Experiments, two SPME fibers were introduced to each vessel and the reported VOC signal of each biological replicate (*n* = 3) per timepoint per condition is the average of *n* = 2 technical replicates (two SPME fibers per vessel). For A + R experiments 1 and 2, SPME timepoints were collected at 48, 72, and 96 h post algae inoculation, and at 72, 96, and 120 h for A + R experiment 3 (as indicated by * in Figure 1). For the freeze–thaw cellular disruptions, one SPME fiber was introduced to sample each vessel, resulting in *n* = 3 biological replicates with a single technical replicate (one SPME fiber) per timepoint per condition. For FTA Experiment 1, only 24 and 48 h SPME sampling timepoints were collected, but for FTA Experiments 2 and 3, SPME sampling was performed at 1, 6, 24, and 48 h timepoints. Additionally, an unexposed fiber representing a “travel blank” to detect extraneous volatiles deposited during the storage process was included with the culture samples. All SPME fibers were subsequently stored at 2–4 °C and analyzed by GC-MS within 2–3 weeks of VOC sample collection. VOC data acquisition utilized an untargeted GC-MS approach performed using an Agilent 5975 T GC-MSD (Agilent Technologies, Santa Clara, CA, USA), employing the same instrument and temperature program used in Reese et al. [24]. Instrument performance was monitored using a commercial reference mixture (S-22329-R1; AccuStandard, New Haven, CT, USA) to evaluate variation in day-to-day performance and to calculate retention indices.

Analytical reference standards for trans-β-ionone (≥97.0% purity, catalogue #16976) and β-cyclocitral (≥97.0% purity, catalogue #16976) were purchased (Sigma-Aldrich, St. Louis, MO, USA) to verify if putative identifications of select biomarkers were accurate. The standards were injected neat (1 µL) into the GC inlet. The analytical standards were analyzed alongside an injection of the commercial reference mixture (AccuStandard) for determination of retention indices values. Resultant mass spectral fragmentations from the analytical standards were then compared to those of experimentally observed biomarkers. The same GCMS method detailed in [24] was also used for these analytical standards except that the analytical standards method required a 4 min solvent delay.

### 4.5. Volatilomics Analysis and Requirements for Defining a ‘Biomarker’ for This Work

Data processing of GC-MS output using Agilent software was conducted similarly to our previous work [24], with select user-defined parameter differences explained here, to isolate, detect, and identify condition-specific VOCs, or putative biomarkers. Briefly, raw ChemStation files were made compatible for use with Agilent’s MassHunter Software. Chemical peaks were isolated and detected via chromatographic deconvolution and visualization using MassHunter Qualitative Analysis (version B.08) with the same parameters as previously used. Mass Profiler Professional (MPP) 12.6.1 software was used to align peaks across samples within a given condition. Putative identification of VOCs was accomplished with spectral searching against the NIST14 mass spectral database, requiring match factors ≥70%. Compounds not exceeding this threshold were annotated using the following nomenclature: Unknown *m/z*##_RI####.

Three criteria were used to classify a detected VOC as a biomarker (Table 1 and Table 2): (1) detection of VOC in a majority of the timepoints within a biological condition within each individual experiment. Accordingly, for the A + R experiments, VOCs were required to be detected in both technical replicates for at least 2 of 3 biological replicates at each sampled timepoint. For the FTA experiments, which included only a single technical replicate, VOCs were required to be present in at least 2 of 3 biological replicates at each sampled timepoint. (2) VOCs passing criteria (1) were then required to be detected in at least 2 out of the 3 repeated experiments. (3) VOCs passing criteria (2) were required to be either (a) absent in the corresponding media blank or travel blank or (b) present in the sample at relative abundances greater than 10× that seen in the media blank or travel blank. VOCs successfully passing the above criteria were subsequently labeled as putative biomarkers. The presence/absence and semi-quantitative comparisons of these putative biomarkers were compared across different experiments (Figure 2).

## 5. Conclusions

**Figure 4:**
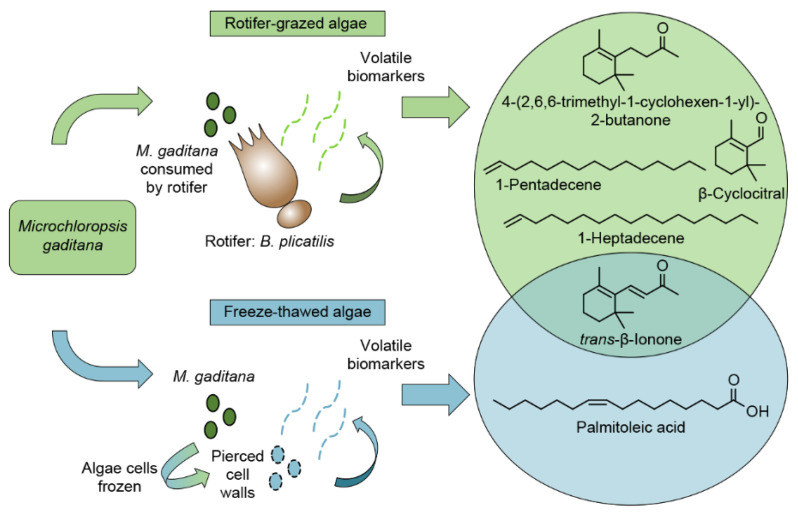
Resulting putative biomarkers from rotifer-grazing and freeze–thawed algal crop damage experiments to *Microchloropsis gaditana* cells.

This work has identified diagnostic biomarkers for biotic and abiotic algal crop damage events (Figure 4). The A + R experiments were designed to allow for the algal consumption by rotifers and the concomitant release of VOCs over the course of days. Conversely, FTA experiments were designed to damage the entire algal culture within a much smaller window of time (e.g., less than 1.5 h), resulting in the immediate release of VOCs characteristic for this damage mechanism. None of the putative biomarkers detected after the algae experienced rotifer-grazing or freeze–thawing events were found in the headspace of healthy algal cultures. Only one biomarker, β-ionone, was common to both A + R and FTA algal crop damage experiments. This suggests that β-ionone, and thus carotenoid degradation might result from algal wounding events, in general, and could be a reliable biomarker of algal crop damage. Our work postulates that the distinct biomarkers generated by different algal crop damage events have potential to not only distinguish healthy from unhealthy ponds, but to further indicate the type of algal crop damage that is underway. Early diagnostic biomarkers of algal crop damage will facilitate more effective and targeted algal pond treatments, thus increasing algal crop yields for subsequent biofuel production.

## Figures and Tables

**Table 1:** Putative biomarkers emitted during grazing of *Microchloropsis gaditana* by *Brachionus plicatilis* and not present in time-matched healthy *M. gaditana* controls.

Compound	Base Peak *m/z*	Tentative Compound Class	NIST14 ID	NIST% Match	Experimental RI	Theoretical RI	A+R Expt 1			A+R Expt 2			A+R Expt 3		
							48 h	72 h	96 h	48 h	72 h	96 h	72 h	96 h	120 h
R1	137	Ketone	β-cyclocitral	83	1208	1220		X	X	X	X	X	X	X	X
R2	148		*unknown*		1402			X		X					
R3	121	Ketone	4-(2,6,6-trimethyl-1-cyclohexen-1-yl)-2-butanone	75	1443	1433					X	X		X	
R4	177	Ketone	β-ionone	80	1494	1486	X	X	X	X	X	X	X	X	X
R5	57	Alkene	1-pentadecene	71	1499	1492	X	X		X			X		
R6	83	Alkene	1-heptadecene	78	1662	1687	X	X		X	X	X	X	X	X

**Table 2:** Putative biomarkers emitted only after freeze–thaw damage to *Microchloropsis gaditana* cultures and not present in time-matched, healthy *M. gaditana* controls.

Compound	Base Peak *m/z*	Tentative Compound Class	NIST14 ID	NIST% Match	Experimental RI	Theoretical RI	FTA Expt 1		FTA Expt 2				FTA Expt 3			
							24 h	48 h	1 h	6 h	24 h	48 h	1 h	6 h	24 h	48 h
F1	193				834				X					X		
F2	119				1177		X	X			X			X	X	X
F3	57				1265		X		X	X			X	X		
F4	79				1288				X				X	X		
F5	81				1294					X			X	X		
F6	85				1381						X				X	X
F7	177	Ketone	β-ionone	93	1495	1486	X		X	X	X		X	X	X	
F8	123				1498		X	X	X	X	X		X	X	X	
F9	55	Carboxylic acid	Palmitoleic acid	80	1954	1951			X				X	X		
F10	149				2821				X					X		

## Data Availability

Sandia National Laboratories retains an archive of the datasets and are available from the corresponding author on reasonable request, because of its usage in the ongoing study.

## Supplementary Materials

The following are available online at https://www.mdpi.com/article/10.3390/metabo11100707/s1, Table S1: VOCs released during grazing of Microchloropsis gaditana by Brachionus plicatilis and not passing strict criteria for classification as putative biomarkers, Table S2: putative biomarkers emitted after freeze–thaw damage to *Microchloropsis gaditana* cultures and not passing the strict criteria for classification as putative biomarkers.
