# Supplementary material for: Abiotic and Biotic Damage of Microalgae Generate Different Volatile Organic Compounds (VOCs) for Early Diagnosis of Algal Cultures for Biofuel Production
Source: Metabolites. 2021 Oct 15;11(10):707. doi: 10.3390/metabo11100707 (PMC8541270; doi:10.3390/metabo11100707)
Supplement: Supplementary file 1 [file metabolites-11-00707-s001.zip › metabolites-1379424-supplementary.pdf]

Supplementary material

Supplemental Table S1. VOCs Emitted During Grazing of *Microchloropsis gaditana* by *Brachionus plicatilis* and Did Not Pass the Strict Criteria for Classification as Putative Biomarkers

Compound	Tentative Compound Class	NIST14 ID	NIST % Match	Base Peak m/z	Experimental RI	Theoretical RI	A+R Expt 1			A+R Expt 2			A+R Expt 3		
							48 h	72 h	96 h	48 h	72 h	96 h	72 h	96 h	120 h
SR1				57	977		A+R	A+R	A+R						
SR2				69	961			A, A+R		A, A+R	A, A+R	A			
SR3	Ketone	2,2,6-trimethyl-cyclohexanone	72	82	1021	1036		A+R	A+R						
SR4				87	1031			A	A						
SR5				167	1390									A	A
SR6				170	1401						A, A+R			A+R	

Note: VOCs present in Algae + Rotifer cultures (A + R), Algae cultures (A), or in both culture types (A, A + R)

Supplemental Table S2. Putative Biomarkers Emitted After Freeze-Thaw Damage to *Microchloropsis gaditana* Cultures and Did Not Pass the Strict Criteria for Classification as Putative Biomarkers

Compound	Tentative Compound Class	NIST14 ID	Base Peak m/z	Experimental RI	Theoretical RI	FTA Expt 1		FTA Expt 2				FTA Expt 3			
						24 h	48 h	1 h	6 h	24 h	48 h	1 h	6 h	24 h	48 h
SF1			41	1048				FTA	FTA	FTA					
SF2			43	848				FTA	FTA						
SF3			43	2146										FTA	FTA
SF4			43	2365									FTA	FTA	FTA
SF5			55	1906		FTA	FTA								
SF6			57	1564								FTA	FTA		
SF7			68	2754		A	A								
SF8			68	899									FTA	FTA	
SF9	Alkene	3,7,11,15-tetramethyl-, [R-[R*,R*-(E)]]-2-Hexadecene	70	1844	1830	A	A								
SF10			71	2117								FTA	FTA	FTA	
SF11			73	1851		A	A								
SF12	Carboxylic Acid	n-Hexadecanoic acid	73	1977	1968	A	A								
SF13			73	1873								FTA	FTA		
SF14			78	766					FTA	FTA					
SF15			79	1513				FTA	FTA						
SF16			79	1289								FTA	FTA		
SF17			79	1582								FTA	FTA		
SF18			79	1647									FTA		
SF19			79	1879									FTA	FTA	
SF20			79	2123								FTA	FTA		
SF21			79	2296								FTA	FTA		
SF22	Diterpene	Neophytadiene	81	1876	1837	A	A								
SF23			83	1649				FTA	FTA						
SF24			101	1393										FTA	FTA
SF25			109	1213					FTA	FTA					
SF26			111	1541		FTA	FTA						FTA	FTA	FTA
SF27	Ketone	Ethanone, 1-(4-methylphenyl)-	119	1176	1183	FTA	FTA						FTA	FTA	FTA
SF28			131	1194					A	A					
SF29			139	1225									FTA		FTA
SF30	Phthalate Ester	Diethyl Phthalate	149	1553	1594	A	A								
SF31			197	1856		FTA	FTA						FTA	FTA	FTA
SF32			207	1725		FTA	FTA								
SF33			220	2104								FTA	FTA		
SF34			239	2147		FTA	FTA								

Note: VOCs present in Freeze-Thaw Damaged cultures (FTA) or Algae cultures (A).
